# The structure of KPN03535 (gi|152972051), a novel putative lipoprotein from *Klebsiella pneumoniae*, reveals an OB-fold

**DOI:** 10.1107/S1744309109018168

**Published:** 2009-10-27

**Authors:** Debanu Das, Piotr Kozbial, Gye Won Han, Dennis Carlton, Lukasz Jaroszewski, Polat Abdubek, Tamara Astakhova, Herbert L. Axelrod, Constantina Bakolitsa, Connie Chen, Hsiu-Ju Chiu, Michelle Chiu, Thomas Clayton, Marc C. Deller, Lian Duan, Kyle Ellrott, Marc-André Elsliger, Dustin Ernst, Carol L. Farr, Julie Feuerhelm, Anna Grzechnik, Joanna C. Grant, Kevin K. Jin, Hope A. Johnson, Heath E. Klock, Mark W. Knuth, S. Sri Krishna, Abhinav Kumar, David Marciano, Daniel McMullan, Mitchell D. Miller, Andrew T. Morse, Edward Nigoghossian, Amanda Nopakun, Linda Okach, Silvya Oommachen, Jessica Paulsen, Christina Puckett, Ron Reyes, Christopher L. Rife, Natasha Sefcovic, Henry J. Tien, Christine B. Trame, Henry van den Bedem, Dana Weekes, Tiffany Wooten, Qingping Xu, Keith O. Hodgson, John Wooley, Ashley M. Deacon, Adam Godzik, Scott A. Lesley, Ian A. Wilson

**Affiliations:** a Joint Center for Structural Genomics, http://www.jcsg.org, USA; b Stanford Synchrotron Radiation Lightsource, SLAC National Accelerator Laboratory, Menlo Park, California, USA; c Program on Bioinformatics and Systems Biology, Burnham Institute for Medical Research, La Jolla, California, USA; d Department of Molecular Biology, The Scripps Research Institute, La Jolla, California, USA; e Center for Research in Biological Systems, University of California, San Diego, La Jolla, California, USA; f Protein Sciences Department, Genomics Institute of the Novartis Research Foundation, San Diego, California, USA; g Photon Science, SLAC National Accelerator Laboratory, Menlo Park, California, USA

**Keywords:** lipoproteins, OB-fold, NipE-like protein, single-stranded DNA-binding proteins, toxins, BOF, human gut pathogens, structural genomics

## Abstract

KPN03535 (gi|152972051) is a putative lipoprotein of unknown function that is secreted by *Klebsiella pneumoniae* MGH 78578. The crystal structure reveals that despite a lack of any detectable sequence similarity to known structures, it is a novel variant of the OB-fold and structurally similar to the bacterial Cpx-pathway protein NlpE, single-stranded DNA-binding (SSB) proteins and toxins. *K. pneumoniae* MGH 78578 forms part of the normal human skin, mouth and gut flora and is an opportunistic pathogen that is linked to about 8% of all hospital-acquired infections in the USA. This structure provides the foundation for further investigations into this divergent member of the OB-fold family.

## Abstract 2

KPN03535 is a protein unique to *K. pneumoniae*. The crystal structure reveals that KPN03535 represents a novel variant of the OB-fold and is likely to be a DNA-binding lipoprotein.

## 1. Introduction

KPN03535 (gi|152972051) is an orphan protein that is exclusively found in *Klebsiella pneumoniae* MGH 78578 (an opportunistic human pathogen belonging to enterbacteriales of gammaproteobacteria; Galperin *et al.*, 2007 ▶; Gill *et al.*, 2006 ▶; Frank & Pace, 2008 ▶; Ley *et al.*, 2008 ▶) and *K. pneumoniae* 342 (three-residue substitution). It consists of 132 residues with a calculated pI of 9.40 and a predicted signal peptide. The N-terminus of KPN03535 has a lipoprotein signature, indicated by the presence of an LSGC motif (von Heijne, 1989 ▶), as well as predictions from *LipoP* 1.0 (Juncker *et al.*, 2003 ▶). It is a singleton protein that has not been assigned to any Pfam family, but sequence-based fold-prediction methods (Ginalski *et al.*, 2003 ▶) suggest similarity to members of the PF01336 family (OB-fold nucleic acid-binding domain). We determined the crystal structure of KPN03535 in order to explore this extremely divergent member of the com­monly occurring OB-fold. Structural comparisons show similarities to the OB-fold-containing Cpx-pathway protein NlpE, single-stranded DNA-binding (SSB) proteins, bacterial OB-fold (BOF) and toxin proteins, which enable inferences about function that may now be tested biochemically. This structure should serve as a basis for understanding structure–function relationships in any newly discovered proteins with a similar sequence, such as those identified by whole microbial genome sequencing and metagenomic surveys of the human microbiome.

## 2. Materials and methods

### 2.1. KPN03535 expression, purification and crystallization

Clones were generated using the Polymerase Incomplete Primer Extension (PIPE; Klock *et al.*, 2008 ▶) cloning method. The gene encoding KPN03535 (gi|152972051; Swiss-Prot A6TEE6) was amplified by polymerase chain reaction (PCR) from *K. pneumoniae* MGH 78578 genomic DNA using *PfuTurbo* DNA polymerase (Stratagene) and I-PIPE (Insert) primers (forward primer, 5′-ctgtacttccagggcGCTTCTAAAGCCTTTTATTCCGCGGGAG-3′; reverse primer, 5′-aattaagtcgcgttaTTTAACCACCTTGGGATTCT­GTAGCGTC-3′; target sequence in upper case) that included sequences for the predicted 5′- and 3′-ends. The expression vector, pSpeedET, which encodes an amino-terminal tobacco etch virus (TEV) protease-cleavable expression and purification tag (MGSDKIHHHHHHEN­LYFQG), was PCR-amplified with V-PIPE (Vector) primers (forward primer, 5′-taacgcgacttaattaactcgtttaaacggtctccagc-3′; reverse primer, 5′-gccctggaagtacaggttttcgtgatgatgatgatgatg-3′). V-PIPE and I-PIPE PCR products were mixed to anneal the amplified DNA fragments together. *Escherichia coli* GeneHogs (Invitrogen) competent cells were transformed with the V-PIPE/I-PIPE mixture and dispensed on selective LB-agar plates. The cloning junctions were confirmed by DNA sequencing. Using the PIPE method, the gene segment encoding residues Met1–Leu22 was deleted for expression of soluble protein as these residues were initially predicted to correspond to either a signal peptide using *SignalP* (Bendtsen *et al.*, 2004 ▶) or trans­membrane helices using *TMHMM*-2.0 (Krogh *et al.*, 2001 ▶). Expression was performed in selenomethionine-containing medium. At the end of fermentation, lysozyme was added to the culture to a final concentration of 250 µg ml^−1^ and the cells were harvested and frozen. After one freeze–thaw cycle, the cells were homogenized in lysis buffer [50 m*M* HEPES pH 8.0, 50 m*M* NaCl, 10 m*M* imidazole, 1 m*M* tris(2-carboxyethyl)phosphine–HCl (TCEP)] and the lysate was clarified by centrifugation at 32 500*g* for 30 min. The soluble fraction was passed over nickel-chelating resin (GE Healthcare) pre-equilibrated with lysis buffer, the resin was washed with wash buffer [50 m*M* HEPES pH 8.0, 300 m*M* NaCl, 40 m*M* imidazole, 10%(*v*/*v*) glycerol, 1 m*M* TCEP] and the protein was eluted with elution buffer [20 m*M* HEPES pH 8.0, 300 m*M* imidazole, 10%(*v*/*v*) glycerol, 1 m*M* TCEP]. The eluate was buffer-exchanged with TEV buffer (20 m*M* HEPES pH 8.0, 200 m*M* NaCl, 40 m*M* imidazole, 1 m*M* TCEP) using a PD-10 column (GE Healthcare) and incubated with 1 mg TEV protease per 15 mg of eluted protein. The protease-treated eluate was passed over nickel-chelating resin (GE Healthcare) pre-equilibrated with HEPES crystallization buffer (20 m*M* HEPES pH 8.0, 200 m*M* NaCl, 40 m*M* imidazole, 1 m*M* TCEP) and the resin was washed with the same buffer. The flowthrough and wash fractions were combined and concentrated for crystallization trials to 16.1 mg ml^−1^ by centrifugal ultrafiltration (Millipore). KPN03535 was crystallized by mixing 100 nl protein solution with 100 nl crystallization solution in a sitting drop over a 50 µl reservoir volume using the nanodroplet vapor-diffusion method (Santarsiero *et al.*, 2002 ▶) with standard Joint Center for Structural Genomics (JCSG; http://www.jcsg.org) crystallization protocols (Lesley *et al.*, 2002 ▶). The crystallization reagent contained 31% polyethylene glycol 600 and 0.1 *M* CHES pH 9.5. No further cryoprotectant was added to the crystal. A cube-shaped crystal with approximate dimensions 80 × 80 × 80 µm was harvested after 42 d at 293 K for data collection. Initial screening for diffraction was carried out using the Stanford Automated Mounting system (SAM; Cohen *et al.*, 2002 ▶) at the Stanford Synchrotron Radiation Lightsource (SSRL; Menlo Park, California, USA). The diffraction data were indexed in the orthorhombic space group *P*2_1_2_1_2_1_. The molecular weight and oligomeric state were determined using a 1 × 30 cm Superdex 200 column (GE Healthcare) in combination with static light scattering (Wyatt Technology). The mobile phase consisted of 20 m*M* Tris pH 8.0, 150 m*M* NaCl and 0.02%(*w*/*v*) sodium azide.

### 2.2. X-ray data collection and structure determination

Single-wavelength anomalous diffraction (SAD) data were collected to 2.46 Å resolution on beamline 9-2 at SSRL at the wavelength corresponding to the peak (λ_1_) of a selenium absorption edge using the *Blu-Ice* data-collection environment (McPhillips *et al.*, 2002 ▶). A data set was collected at 100 K using a MAR Mosaic 325 CCD detector (Rayonix USA). The SAD data were integrated and reduced using *MOSFLM* (Leslie, 1992 ▶) and scaled with the program *SCALA* (Collaborative Computational Project, Number 4, 1994 ▶). Phasing was performed with *SHELXD* (Sheldrick, 2008 ▶) and *autoSHARP* (Vonrhein *et al.*, 2007 ▶) [20 selenium sites per asymmetric unit, overall FOM (acentric/centric) = 0.34/0.12, overall phasing power (anomalous differences) = 1.2] and automated iterative model building was performed with *RESOLVE* (Terwilliger, 2003 ▶). Model completion and crystallographic refinement were performed with *Coot* (Emsley & Cowtan, 2004 ▶) and *REFMAC*5 (Collaborative Computational Project, Number 4, 1994 ▶) with TLS (one group per monomer) refinement (Winn *et al.*, 2003 ▶) and medium NCS restraints for all chains. Data and refinement statistics are summarized in Table 1 ▶.

**Figure 1:**
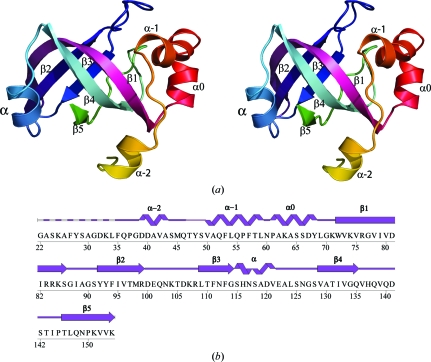
Crystal structure of KPN03535. (*a*) Stereo ribbon representation of the KPN03535 monomer color-coded from the N-terminus (yellow) to the C-terminus (green). The nomenclature for helix α and strands β1–β5 follows that used for the OB-fold (Murzin, 1993 ▶). Helices α−2, α−1 and α0 are unique to KPN03535. (*b*) Diagram showing the secondary-structure elements of KPN03535 superimposed on the primary amino-acid sequence. Helices and β-strands are indicated. The protein was expressed with a purification tag that was removed, leaving a residual Gly residue at the N-terminus followed by the KPN03535 sequence.

The quality of the crystal structure was analyzed using the JCSG Quality Control server, which verifies the stereochemical quality of the model using *AutoDepInputTool* (Yang *et al.*, 2004 ▶), *MolProbity* (Davis *et al.*, 2004 ▶) and *WHATIF* 5.0 (Vriend, 1990 ▶), the agreement between the atomic model and the data using *SFCHECK* 4.0 (Vaguine *et al.*, 1999 ▶) and *RESOLVE* (Terwilliger, 2000 ▶), the protein sequence using *ClustalW* (Thompson *et al.*, 1994 ▶), atom occupancies using *MOLEMAN*2 (Kleywegt, 2000 ▶) and the consistency of NCS pairs. This analysis also evaluates difference in *R*_cryst_/*R*_free_, expected *R*_free_/*R*_cryst_ and maximum/minimum *B* values by parsing the refinement log-file and PDB header. Protein quaternary structure analysis was performed using the *PISA* server (Krissinel & Henrick, 2005 ▶). Fig. 1 ▶(*b*) was adapted from an analysis using *PDBsum* (Laskowski *et al.*, 2005 ▶) and all other figures were prepared with *PyMOL* (DeLano, 2002 ▶). Atomic coordinates and experimental structure factors for KPN03535 have been deposited in the PDB under accession code 3f1z. A systematic search for other proteins of similar structure was conducted using several different methods including the *DALI* server (Holm *et al.*, 2008 ▶), the protein structure comparison service *SSM* at the European Bioinformatics Institute (http://www.ebi.ac.uk/msd-srv/ssm; Krissinel & Henrick, 2004 ▶) and the flexible structure-alignment method *FATCAT* (Ye & Godzik, 2003 ▶).

## 3. Results and discussion

### 3.1. Overall structure

Residues 1–22 of the full-length protein (1–154) were initially predicted to represent a signal peptide and were removed during cloning. The crystallized protein is comprised of a glycine left after cleavage of the expression and purification tag followed by KPN03535 residues 23–154. The final model contains ten monomers (chains *A*–*J*), two PEG molecules (PEG 600 fragments from the crystallization solution) and 323 water molecules in the asymmetric unit. The ten monomers are almost identical in structure and completeness and superimpose extremely well, with pairwise r.m.s.d. values ranging from only 0.2 to 0.4 Å. Residues 23–35 in chains *A* *B*, *C*, *E* and *J*, 23–36 and 154 in chain *D*, 23–35 and 154 in chain *F*, 23–36 in chains *G* and *H*, and 23–38 in chain *I* and the N-terminal glycine in all chains are disordered and have not been modeled. The Matthews coefficient (Matthews, 1968 ▶) is ∼3.2 Å^3^ Da^−1^, with an estimated solvent content of ∼62%. The Ramachandran plot produced by *MolProbity* (Davis *et al.*, 2004 ▶) shows that 98.5% and 100% of amino acids are in the favored and allowed regions, respectively.

**Figure 2:**
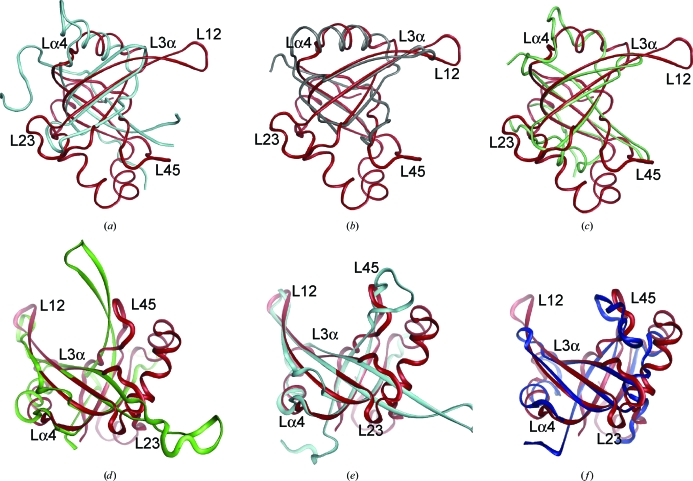
Superimposition of the crystal structure of KPN03535 (red) on OB-fold proteins that have N-terminal lipoprotein sequence, such as (*a*) NlpE, (*b*) shiga toxin and (*c*) BOF, and single-stranded DNA-binding proteins (SSBs), such as (*d*) *E. coli* SSB, (*e*) *E. coli* PriB and (*f*) *T. thermophilus* aspartyl-tRNA synthetase.

Residues 70–154 of the monomer form the OB-fold comprised of a five-stranded β-sheet (β1, β2, β3, β4 and β5) capped by a short α-­helix (α) based on the standard OB-fold nomenclature (Murzin, 1993 ▶; Fig. 1 ▶ *a*). The capping helix is shorter than those observed in most other OB-fold proteins (Fig. 2 ▶). Residues 36–69 constitute three additional α-helices (α−2, α−1 and α0) which are not observed in other structures of the same fold. The curved β-sheet forming the β-­barrel core of the OB-fold is highly conserved in size and structure,  while the largest variations are seen in the three loops (L_12_, L_23_ and L_45_) that extend in different directions from the core and are often functionally important.

Crystal-packing and assembly analysis using *PISA* (Krissinel & Henrick, 2005 ▶) supported by analytical size-exclusion chromatography and static light scattering suggest that a monomer is the likely oligomeric state. In the crystal structure, the protein assembles as two stacked pentameric rings, formed by loose interdigitation of the ‘finger-like’ β1–L_12_–β2 structure, with outer and inner diameters of ∼80 Å and ∼40 Å, respectively, and a thickness of ∼40 Å. The buried surface area of each monomer within each pentamer (∼540 Å^2^) and each monomer in the interface between the two pentamers (∼600 Å^2^) is low. The quaternary structure analysis does not suggest sufficiently strong and extensive interactions to enable complex formation in solution, suggesting that these pentamers could be a crystallization artifact. The N-terminus of each monomer extends into the solvent and probably does not have an impact on the oligomerization state. In the absence of any biochemical data, the functional oligomeric state of the protein remains unknown.

### 3.2. Functional hypotheses

#### 3.2.1. NlpE-like

The only other reported bacterial lipoprotein containing an OB-fold is the C-terminal domain of *E. coli* NlpE (new lipoprotein E), which is an outer membrane lipoprotein in Gram-negative bacteria involved in the envelope stress response in the Cpx pathway. It activates the Cpx, two-component, signal transduction pathway composed of the inner membrane histidine kinase CpxA and the cytoplasmic response regulator CpxR (Raivio & Silhavy, 1997 ▶). The Cpx pathway controls the production of the periplasmic protease DegP and other proteins involved in fighting cellular stress (Snyder *et al.*, 1995 ▶; Danese *et al.*, 1995 ▶; Raivio *et al.*, 1999 ▶). Other proteins are also implicated in the regulation of the Cpx pathway. For example, CpxP with an LT*XX*Q motif (Pfam PF07813) is involved in feedback inhibition of the Cpx pathway (Danese *et al.*, 1998 ▶; Danese & Silhavy, 1998 ▶). In *K. pneumoniae*, a periplasmic CpxP-like protein with the LT*XX*Q motif, KPN03534, is the neighboring gene to KPN03535. Therefore, KPN03535, like KPN03534, may play a role in the Cpx pathway, similar to NlpE. KPN03535 superimposes fairly well on *E. coli* NlpE (PDB code 2z4i; Hirano *et al.*, 2007 ▶; r.m.s.d. = 3.3 Å, 16% sequence identity, *Z* score 2.3; Fig. 2 ▶ *a*). Despite extremely low sequence identity, some residues are conserved in KPN03535 (Arg76, Asp100, Thr105, Lys107, Arg108 and Asn117) from structure-based sequence alignment. However, the functional roles of these residues in NlpE are not known.

#### 3.2.2. Toxin and BOF-like

Other bacterial OB-fold proteins that have an N-terminal signal sequence are toxins, such as the shiga toxin (PDB code 1r4p; Fraser *et al.*, 2004 ▶; Fig. 2 ▶ *b*), cholera toxin (PDB code 3efx; Holmner *et al.*, 2004 ▶; r.m.s.d. = 2.8 Å, 6% sequence identity, *Z* score 5.8) and a bacterial OB-fold (BOF; Ginalski *et al.*, 2004 ▶) protein (1nnx; O. Lehmann, A. Galkin, S. Pullalarevu, E. Sarikaya, W. Krajewski, K. Lim, A. Howard & O. Herzberg, unpublished work; r.m.s.d. = 2.9 Å, 14% sequence identity; *Z* score 7.4; Fig. 2 ▶ *c*). Neither NlpE nor the toxins have all three of the N-­terminal helices (α0, α−1, α−2) found in KPN03535, but α−2 is observed in cholera toxin (3efx) and α−1 is observed in BOF protein (1nnx). The capping helix α in KPN03535 is shorter than in the toxins and NlpE, although it is similar to that observed in BOF protein. The β-strands forming the curved β-barrel in all these structures are of similar length, but with differences in the loop sizes that connect the β-­strands.

#### 3.2.3. Single-stranded DNA-binding protein, SSB-like

**Figure 3:**
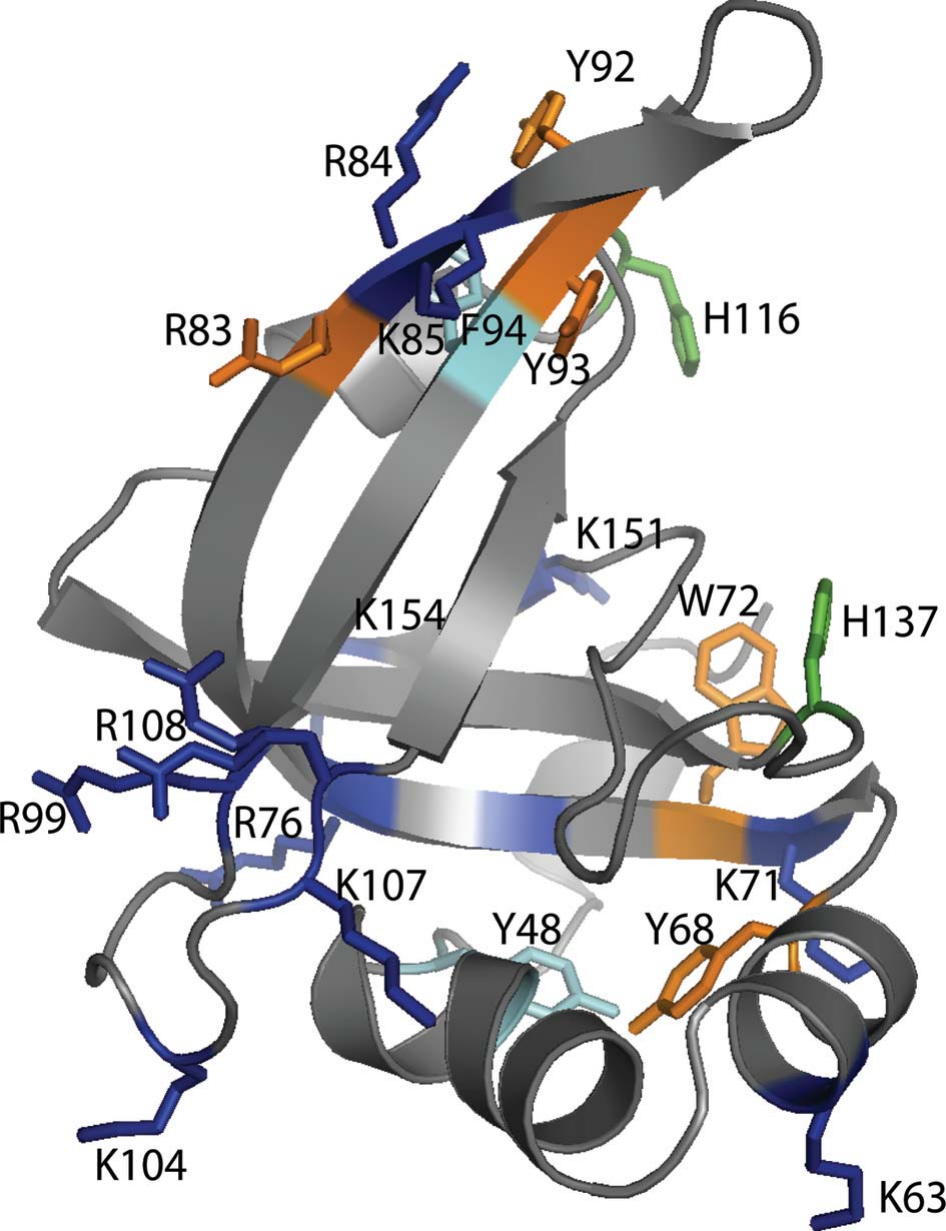
Surface-exposed charged and aromatic residues on KPN03535 that may be functionally important if this protein binds DNA or RNA (for clarity, the view of the monomer shown here is different from that shown in Fig. 4 ▶ and was obtained by a 180° rotation around a horizontal axis followed by a 180° rotation around a vertical axis). Arg83, Arg84 and Lys85 comprise the positive surface region described in Fig. 4 ▶. Arg84 and Lys85 are conserved as Arg17 and Lys18 in the *E. coli* PriB structure and as Arg29 in *T. thermophilus* aspartyl-tRNA synthetase. Phe94 is conserved in shiga toxin, but there are currently no reports of any functional role of this residue in the toxin.

Single-stranded DNA-binding proteins (SSBs) also possess OB-folds and are involved in a multitude of cellular functions, such as DNA replication, transcription, recombination, repair, translation, cold-shock response and maintenance of telomeres (Theobald *et al.*, 2003 ▶; Chase & Williams, 1986 ▶; Wold, 1997 ▶; Meyer & Laine, 1990 ▶; Lohman & Ferrari, 1994 ▶; Lohman *et al.*, 1996 ▶). KPN03535 is structurally similar to OB-fold SSBs, including *E. coli* SSB (PDB code 1eyg; Raghunathan *et al.*, 2000 ▶; r.m.s.d. 2.7 Å; 13% sequence identity; *Z* score 7.0; Fig. 2 ▶ *d*), *E. coli* PriB (PDB code 1v1q; Liu *et al.*, 2004 ▶; r.m.s.d. 2.3 Å; 13% sequence identity, *Z* score 8.0; Fig. 2 ▶ *e*), *Thermus thermophilus* aspartyl-tRNA synthetase (PDB code 1l0w; Ng *et al.*, 2002 ▶; r.m.s.d. 2.6 Å; 11% sequence identity; *Z* score 9.0; Fig. 2 ▶ *f*) and human mitochondrial SSB (PDB code 3ull; Yang *et al.*, 1997 ▶; r.m.s.d. 2.7 Å; 8% sequence identity, *Z* score 7.1). The N-terminal α−1 and α0 secondary-structure elements in KPN03535 are partially conserved in aspartyl-tRNA synthetase, but not in the other structures. Many of the loops in OB-fold ssDNA-binding proteins are either involved in interactions with DNA or in quaternary interactions that result in the various oligomeric forms. For example, loop L_45_, which makes the most interactions with DNA in PriB (Huang *et al.*, 2006 ▶) and aspartyl-tRNA synthetase, is similar to that of KPN03535, but is much longer in *E. coli* and in human mitochondrial SSBs. Among the surface-exposed Arg, Lys and aromatic residues that could be functionally relevant if KPN03535 were to bind DNA or RNA (Fig. 3 ▶), Arg84 and Lys85 of KPN03535 are conserved and correspond to Arg17 and Lys18 in PriB, where Lys18 is involved in ssDNA-binding (Huang *et al.*, 2006 ▶). Arg83 and Arg99 of KPN03535 are conserved in aspartyl tRNA synthetase as Arg29 (equivalent to Arg28 in the *E. coli* aspartyl-tRNA synthetase that binds to tRNA; Eiler *et al.*, 1999 ▶) and Arg39. Multiple structural alignment of various OB-fold proteins using the *POSA* method (Ye & Godzik, 2005 ▶) suggests that KPN03535 has a closer relationship to tRNA synthetases than to the BOF protein and is most distant from OB-fold toxins.

**Figure 4:**
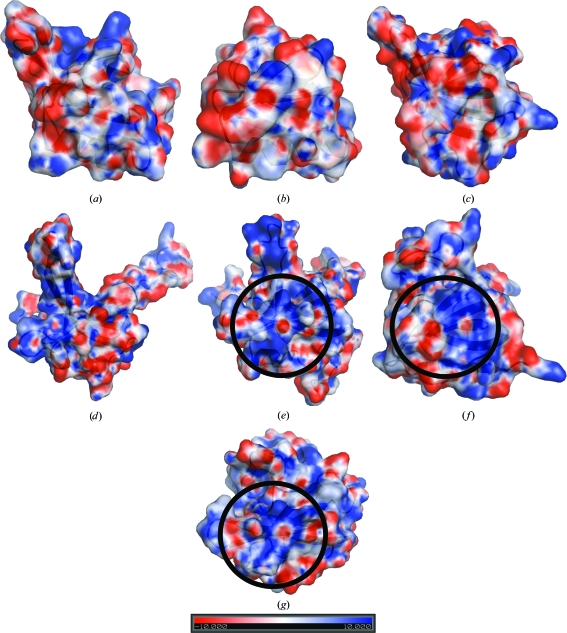
Comparison of the electrostatic surface potentials of monomers of (*a*) NlpE, (*b*) shiga toxin, (*c*) BOF, (*d*) *E. coli* SSB, (*e*) *E. coli* PriB, (*f*) *T. thermophilus* aspartyl-tRNA synthetase and (*g*) KPN03535. All the figures are in approximately the same orientation and reflect the surface view that would be presented for oligonucleotide binding, as in tRNA synthetase. The figure reveals that the positively charged surface patch (central blue portion in black circles) on the KPN03535 most closely resembles that of *E. coli* PriB and is also similar to that seen in aspartyl-tRNA synthetase. In KPN03535, this positively charged patch is formed by Arg83, Arg84 and Lys85. The corresponding conserved residues are Arg17 and Lys18 in PriB and Arg29 in aspartyl-tRNA synthetases, respectively.

Analysis of the electrostatic surface potential indicates that KPN03535 most closely resembles PriB and aspartyl-tRNA synthetase (Fig. 4 ▶), with a prominent positively charged area similar to the DNA-binding region of these two proteins. Interestingly, this patch is different from that observed in the *E. coli* SSB, which reflects the known differences in ssDNA-binding modes of SSB and PriB. The basic nature of KPN03535 (pI 9.4) also hints at the possibility of oligonucleotide binding.

In conclusion, the crystal structure of KPN03535 reveals a novel divergent member of the prevalent OB-fold and suggests that it is most likely to be a nucleic acid-binding protein. As for the recently solved structure of MPN554 from *Mycoplasma pneumoniae* (Das *et al.*, 2007 ▶), another novel OB-fold with unknown cellular function but with single-stranded DNA-binding properties, the structure of KPN03535 reveals that further exploration of the functionality of the OB-fold is necessary. Bacterial lipoproteins have many important functions and are potential vaccine candidates (Steere *et al.*, 1998 ▶; Myers *et al.*, 2007 ▶). *K. pneumoniae* is an opportunistic pathogen that is prevalent in immunocompromised patients in hospitals and in patients with liver disease (Hidron *et al.*, 2008 ▶; Pope *et al.*, 2008 ▶). Functional inferences that can be drawn from this crystal structure should now allow focused structure-assisted biochemistry to establish the exact molecular and cellular role for this protein.

Additional information about KPN03535 is available from TOPSAN (Krishna *et al.*, 2010 ▶)  http://www.topsan.org/explore?PDBid=3f1z.

## Supplementary Material

PDB reference: KPN03535, 3flz, r3f1zsf

## Figures and Tables

**Table 1:** Crystallographic data and refinement statistics for KPN03535 (PDB code 3f1z) Values in parentheses are for the highest resolution shell.

Space group	*P*2_1_2_1_2_1_
Unit-cell parameters (Å)	*a* = 97.42, *b* = 105.51, *c* = 181.25
Data collection	
Wavelength (Å)	0.9792 [Se peak (λ_1_)]
Resolution range (Å)	29.9–2.46 (2.52–2.46)
No. of observations	509996
No. of unique reflections	68362
Completeness (%)	99.8 (99.7)
Mean *I*/σ(*I*)	15.4 (2.5)
*R*_merge_ on *I*† (%)	11.1 (69.6)
Model and refinement statistics	
Resolution range (Å)	29.9–2.46
No. of reflections (total)	68310‡
No. of reflections (test)	3458
Completeness (%)	99.7
Data set used in refinement	λ_1_
Cutoff criterion	|*F*| > 0
*R*_cryst_§	0.192
*R*_free_§	0.228
Stereochemical parameters	
Restraints (r.m.s.d. observed)	
Bond angle (°)	1.70
Bond length (Å)	0.017
Average isotropic *B* value (Å^2^)	38.2¶
ESU†† based on *R*_free_ (Å)	0.22
Protein residues/atoms	1182/9162
Water/PEG molecules	323/2

† *R*_merge_ = 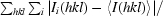 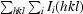.

‡ Typically, the number of unique reflections used in refinement is slightly less than the total number that were integrated and scaled. Reflections are excluded owing to systematic absences, negative intensities and rounding errors in the resolution limits and unit-cell parameters.

§ *R*_cryst_ = 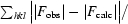 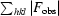, where *F*_calc_ and *F*_obs_ are the calculated and observed structure-factor amplitudes, respectively. *R*_free_ is as for *R*_cryst_, but for 5.1% of the total reflections chosen at random and omitted from refinement.

¶ This value represents the total *B* that includes TLS and residual *B* components.

†† ESU, estimated overall coordinate error (Collaborative Computational Project, Number 4, 1994 ▶; Tickle *et al.*, 1998 ▶).
